# Association Between ABCA1 Gene Polymorphisms and the Risk of Hypertension in the Chinese Han Population

**DOI:** 10.3389/fpubh.2022.878610

**Published:** 2022-05-20

**Authors:** Yanli Ren, Enyu Tong, Chunhong Di, Yunheng Zhang, Liangwen Xu, Xiaohua Tan, Lei Yang

**Affiliations:** ^1^Medical School, Hangzhou Normal University, Hangzhou, China; ^2^Affiliated Hospital, Hangzhou Normal University, Hangzhou, China

**Keywords:** hypertension, ABCA1, association, gene polymorphisms, Chinese

## Abstract

**Background:**

Hypertension is rising as a major public health burden around the world. This study explored the association between single-nucleotide polymorphisms (SNPs) in the adenosine triphosphate (ATP)-Binding Cassette Subfamily A1 (ABCA1) gene and hypertension among Chinese Han adults.

**Method:**

A total of 2,296 Han Chinese in southeast China were recruited for this study. We collected medical reports, lifestyle details, and blood samples from individuals. The polymerase chain reaction-ligase detection reaction (PCR-LDR) method was used to detect the genotypes of these SNPs in the ABCA1 gene.

**Results:**

After adjusting some covariates, the additive and recessive models of the rs2472510 and rs2515614 were significantly associated with hypertension. The haplotypes TCTA (rs2297406-rs2472433-rs2472510-rs2515614) were associated with high SBP, and the haplotypes CCTA, TCTA, and TTTA were associated with high diastolic blood pressure (DBP).

**Conclusion:**

The results of the relationship between the polymorphisms of rs2297406, rs2472433, rs2472510, and rs2515614 in ABCA1 and hypertension in southeastern China would provide a theoretical basis for genetic screening and disease prevention.

## Introduction

Hypertension is becoming a major public health burden around the world. The latest data from the World Health Organization (WHO) estimated that 1.28 billion adults who aged 30–79 years worldwide have hypertension, over 1 billion people lived in low- and middle-income countries in 2019 (1). Essential hypertension (EH) is the most common type that affected 85% of those with hypertension (2). In addition, it is a major risk of cardiovascular and cerebrovascular diseases, such as angina, heart attack, stroke, heart failure, and kidney failure (3–7). Modifiable environmental elements of hypertension include unhealthy diets, physical inactivity, consumption of tobacco and alcohol, and obesity (8–10). Furthermore, genetic factors should play a key role in the etiology of hypertension and making an effect on cardiovascular diseases (11, 12).

Adenosine triphosphate-binding cassette transporter A1 (*ABCA1*) belongs to the ABC gene family, and the human *ABCA1* gene has a total length of 149 kb that includes a 14,553-bp promoter, 146,581 bp of intron and exons, and a 1-kb 3′ flanking region (13). Intracellular cholesterol metabolic homeostasis is maintained by a complex network, regulating cholesterol biosynthesis, uptake, efflux, conversion, esterification, and cholesterol trafficking (14). The level of cholesterol in the inner leaflet of the plasma membrane was exactly controlled by the *ABCA1*, the main participant in cholesterol efflux (15). Therefore, loss of *ABCA1* function could affect the cholesterol levels. Some research studies proposed that the abnormal ABCA1 cholesterol transporting function may cause hypertension by stimulating epithelial sodium channels (ENaCs), the vital components in the regulation of total body Na+ homeostasis, and might also lead to the occurrence of hypertension (16). In addition, Xu et al. surveyed that the expression level of the *ABCA1* gene was significantly decreased in hypertensive patients who were newly diagnosed but untreated; Yamada et al. found that the −14C->T polymorphism of the ABCA1 gene had been appreciably affiliated with the prevalence of hypertension in Japanese individuals (17, 18).

Therefore, we performed a case-control study of the Chinese Han population to estimate the risk of hypertension with polymorphisms of *ABCA1* in Zhejiang province and predict the genetic risk of this condition.

## Method

### Subjects

This study adopted a cluster random sampling method and randomly selected four townships in Ningbo City, Zhejiang Province. From April 2013 to July 2013, health was examined at these four townships (Hengjie, Gaoqiao, Gulin, and Jishigang) health service centers for persons ≥40 years of age. The inclusion criteria of this study were those who were 1) willing to participate in this study and signed an informed consent form, 2) aged 40 or above, and 3) Han Chinese. The exclusion criteria were as follows: 1) not signed the informed consent form, 2) under the age of 40, 3) other ethnic minorities in China, and 4) missing information during the data collection process. The final population included in the analysis was 2,296 individuals.

### Diagnostic Criteria

The definition of hypertension refers to the “China Guidelines for the Prevention and Treatment of Hypertension (2018 Revised Edition)” that is compiled by the China Hypertension Prevention and Control Guidelines Revision Committee, and systolic blood pressure (SBP) greater than or equal to 140 mmHg or diastolic blood pressure (DBP) greater than or equal to 90 mmHg was determined as hypertension (19).

### Epidemiological Investigation

The field of epidemiological investigation mainly adopts the method of a face-to-face interview. The survey content mainly includes general demographic information (such as name, gender, age, education level, etc.), lifestyle behaviors (such as smoking, drinking, dietary information, physical exercise, etc.), and physical examination (such as height, weight, waist circumference, etc.). Smoking history was defined as having smoked one or more cigarettes per day in the past 1 year or more. Drinking history was defined as the average daily consumption of white wine ≥50 g (1 tael), red wine ≥150 g (3 taels), or beer ≥ 500 g (1 catty) for more than 1 year.

The physical examination requirements were as follows: (1) Blood pressure (BP) measurement: BP of each respondent was measured after standing for 5 min. The measurement time interval was not less than 30 s, and the average value was recorded. (2) Height measurement: During the measurement, the subject stood upright with the head, shoulders, hips, and heels close to the measuring ruler. (3) Waist circumference measurement: During the measurement, the subject stood upright with the abdomen in a natural posture, and the tape measure was close to the skin to measure the narrowest part of the waist. (4) Weighing: Before weighing, each respondent takes off his coat and wears only necessary underwear. All measurements were performed three times and the average value was recorded. (5) Body mass index (BMI) was recorded as follows: weight (kg)/height squared (m^2^), and taking 18.5 < BMI < 24 as normal weight, 24 ≤ BMI < 28 as overweight, and BMI ≥ 28 as obesity.

### Single-Nucleotide Polymorphism (SNP) Selection and Genotyping

A total of 2,429 blood samples were collected from the antecubital vein after the subjects had fasted for ≥8 h. Part of the collected samples was used to examine biochemical indicators, such as serum lipid levels, whereas the other part was transferred into a test tube containing anti-coagulant solution to extract DNA. Peripheral venous blood was collected by a professional nurse and all blood samples were stored at −80°C. The phenol-chloroform method was used to extract DNA from peripheral blood leukocytes. The DNA concentration was determined by spectrophotometry, and the DNA concentration was diluted to 10–30 ng/μl, which was used as a template for polymerase chain reaction (PCR) amplification. The PCR reaction volume contained 1 μl of DNA, 1.5 μl of 10× buffer, 1.5 μl of MgCl2, 0.3 μl of dNTPs, 0.15 μl of each primer, 0.2 μl of Taq enzyme, and water to make the total volume 15 μl. The amplification conditions were as follows: 94°C for 3 min; 35 cycles of denaturation at 94°C for 15 s, annealing at 55°C for 15 s, and extension at 72°C for 30 s; and 72°C for 3 min. The ligation reaction volume comprised of 3 μl PCR product, 1 μl of 10× Taq DNA ligase buffer, 0.125 μl of Taq DNA ligase (40 U/μl), 0.01 μl of each discriminating probe, and water to make the total volume 10 μl. The reaction conditions were as follows: 30 cycles of 94°C for 30 s and 56°C for 3 min. To 1 μl of the extension product, 8 μl of loading buffer was added, followed by denaturation at 95°C for 3 min. Next, the reaction products were detected by electrophoresis, and the mixture was analyzed using a sequencer (ABI 3730XL). The polymerase chain reaction-ligase detection reaction (PCR-LDR) method was employed for genotyping detection. PCR was used to amplify the gene fragment containing the mutation site, and three probes were designed based on the SNP site to distinguish the genotype of the site. Two of the probes were completely complementary to the upstream sequence of the SNP site, except that they had different lengths, and the 3'end bases were different according to the genotype. The other probe (FAM fluorescently labeled) was complementary to the downstream sequence of the SNP. According to the characteristics of the ligase, the length of the ligated fragments obtained from different genotypes was different. GeneMapper software was used to analyze the data of the ligated fragments to obtain different genotypes. Primers, probe sequences, and product lengths for genotyping at each site are shown in Appendix Table 1.

Single nucleotide polymorphisms were mainly searched using the PubMed and GeneCards databases. The specific screening process was as follows: (1) literature related to gene polymorphisms and hypertension was searched in NCBI-PubMed, and SNPs were screened; (2) GeneView information was obtained for relevant SNPs from the GeneCards and NCBI databases, and then, missense mutations, 3′ untranslated region (3′ UTR), 5′UTR, or transcription factor-binding sites were selected. (3) The minor allele frequency (MAF) of SNPs in the Chinese population was detected from the HapMap database for the international human genome, and SNP sites with MAF values greater than 0.05 were screened.

### Statistical Analysis

The genetic model analysis is one of the commonly used analysis methods to analyze the association between different alleles and diseases and is the primary association analysis method to determine how SNPs affect disease risk. Genetic models include additive models, dominant models, and recessive models. There were three possible genotypes for a major gene with two alleles: aa, aA, and AA, then if Y is the phenotype and G is the genotype, then the penetrance is expressed as Pr (Y | G) (penetrance function is a set of probability functions of phenotype to genotype), then the dominant genetic model is Pr(Y|aA) = Pr(Y|AA), and the recessive genetic model is Pr(Y)|aA) = Pr(Y |aa), the additive genetic model is Pr (Y | aA) at a position between Pr (Y | AA) and Pr (Y | aa). Statistical analysis was performed using SPSS 24.0 software (SPSS Inc., Chicago, IL, USA). The relationship between demographic information and hypertension was assessed using the χ^2^ test and *t-*test. The Person χ^2^ test was used to determine whether the distribution of points in the population conformed to the Hardy-Weinberg Equilibrium law. The genetic balance was considered to be achieved with *p* > 0.05, and inheritance was stable in the population. A binary logistic regression model was used to analyze the relationship between ABCA1 gene polymorphisms and hypertension. The forest map used the R language (package “forestplot”). Haploview 4.2 software was used for haplotype analysis. *p* < 0.05 indicated that the difference was statistically significant.

## Results

### Basic Characteristics of the Surveyed Population and Analysis of Factors Related to Hypertension

A total of 2,296 people were included in this study. Table 1 shows the basic characteristics of the respondents that include 1,050 men and 1,246 women. The age of the study population was 40 years or older and some blood indicators and lifestyle factors were also included in the table. Then, according to the classification criteria for hypertension, the population was divided into a case group and a control group, of which 972 (42.33%) had high systolic blood pressure (SBP) and 690 (30.05%) had high diastolic blood pressure (DBP). Table 2 shows the relationship between hypertension and the various factors analyzed. In the high SBP group, age, BMI, waist circumference, TC/TG/LDL/HDL, family history, education level, daily iodized salt consumption, and smoking history were associated with high SBP in this study population, while sex, frequency of eating fried foods weekly, frequency of eating dessert weekly, drinking history, and frequency of weekly exercises were not associated with high SBP. Sex, age, BMI, waist circumference, TC/TG/LDL, family history, education level, frequency of eating fried foods weekly, frequency of eating desserts weekly, and daily iodized salt consumption were associated with high DBP in this study population, whereas HDL level, smoking history, drinking history, and frequency of weekly exercise were not statistically different in the high DBP group. Simultaneously, the Hardy–Weinberg Equilibrium analysis was performed on the selected SNPs. The results show that rs2472510, rs2515614, rs2297406, and rs2472433 reach genetic equilibrium in the sample population of this study (Table 3).

**Table 1:** Demographic characteristics.

**Characteristic**		**People who were surveyed**	
		**n**	**%**
Sex	Male	1,050	45.73
	Female	1,246	54.27
Age	40–50	589	25.65
	51–60	617	26.87
	61–70	660	28.75
	Over 70 years old	430	18.73
BMI	Normal	1,441	62.76
	Overweight	693	30.18
	Obesity	162	7.06
Waist, mean (SD)		81.37	8.32
TC, mean (SD)		4.89	0.92
TG, median (IQR)		1.28	0.82
LDL-C, mean (SD)		3.07	0.81
HDL-C, mean (SD)		1.3	0.31
Family history	No	1,770	77.09
	Hypertension	499	21.73
	Hyperlipidemia	27	1.18
Education level	Primary education or below	1,650	71.86
	Secondary education	555	24.17
	High school education or above	91	3.96
Eat fried foods weekly	No	915	39.85
	1–4 times	1,309	57.01
	More than 5 times	72	3.14
Eat dessert weekly	No	729	31.75
	1–4 times	1,418	61.76
	More than 5 times	149	6.49
Daily iodized salt consumption	3g or less	105	4.57
	4–6 g	1,437	62.59
	7g or above	754	32.84
Smoking history	No	1,623	70.69
	Yes	673	29.31
Drinking history	No	1,558	67.86
	Yes	738	32.14
Exercise weekly	No	1,492	64.98
	1–3 times	375	16.33
	4–7 times	429	18.68

*BMI, body mass index; SD, standard deviation; IQR, interquartile range; TC, total cholesterol; TG, triglyceride; HDL-C, high-density lipoprotein cholesterol; LDL-C, low-density lipoprotein cholesterol*.

**Table 2:** General characteristics and lifestyle factors between case and control groups.

**Variable**		**Normal SBP**		**High SBP**		**χ2/t**	***P***	**Normal DBP**		**High DBP**		**χ2/t**	***P***
		***n***	**%**	***n***	**%**			***n***	**%**	***n***	**%**		
Sex	Male	680	60.61	442	39.39	0.045	0.831	708	67.43	342	32.57	5.841	0.016
	Female	716	57.46	530	42.54			898	72.07	348	27.93		
Age	40–50	472	80.14	117	19.86	256.047	<0.001	463	78.61	126	21.39	38.825	<0.001
	51–60	386	62.56	231	37.44			441	71.47	176	28.53		
	61–70	325	49.24	335	50.76			417	63.18	243	36.82		
	Over 70 years old	141	32.79	289	67.21			285	66.28	145	33.72		
BMI	Normal	920	63.84	521	36.16	71.252	<0.001	1,097	76.13	344	23.87	77.862	<0.001
	Overweight	346	49.93	347	50.07			427	61.62	266	38.38		
	Obesity	58	35.8	104	64.2			82	50.62	80	49.38		
Waist, mean (SD)		80.16 ± 7.90		83.03 ± 8.58		−8.298	<0.001	80.34 ± 8.10		83.77 ± 8.33		−9.098	<0.001
TC, mean (SD)		4.77 ± 0.89		5.06 ± 0.94		−7.342	<0.001	4.81 ± 0.91		5.09 ± 0.93		−6.663	<0.001
TG, mean (SD)		1.39 ± 0.79		1.69 ± 1.02		−7.975	<0.001	1.43 ± 0.81		1.72 ± 1.08		−7.299	<0.001
LDL, mean (SD)		2.98 ± 0.79		3.18 ± 0.83		−5.911	<0.001	2.99 ± 0.80		3.24 ± 0.83		−6.603	<0.001
HDL, median (IQR)		1.27 (0.38)		1.23 (0.35)		2.551	0.011	1.26 (0.37)		1.24 (0.35)		0.815	0.415
Family history	No	1,121	63.33	649	36.67	119.568	<0.001	1,317	74.41	453	25.59	74.94	<0.001
	Hypertension	182	32.91	371	67.09			271	54.31	228	45.69		
	Hyperlipidemia	21	77.78	6	22.22			18	66.67	9	33.33		
Education level	Primary education or below	860	52.12	790	47.88	74.207	<0.001	1,115	67.58	535	32.42	15.739	<0.001
	Secondary education	396	71.35	159	28.65			421	75.86	134	24.14		
	High school education or above	68	74.73	23	25.27			70	76.92	21	23.08		
Eat fried foods weekly	No	513	56.07	402	43.93	2.242	0.326	668	73.01	247	26.99	6.85	0.033
	1–4 times	772	58.98	537	41.02			888	67.84	421	32.16		
	More than 5 times	39	54.17	33	45.83			50	69.44	22	30.56		
Eat dessert weekly	No	408	55.97	321	44.03	3.669	0.16	540	74.07	189	25.93	11.929	0.003
	1–4 times	820	57.83	598	42.17			955	67.35	463	32.65		
	More than 5 times	96	64.43	53	35.57			111	74.5	38	25.5		
Daily iodized salt consumption	3g or less	79	75.24	26	24.76	84.713	<0.001	88	83.81	17	16.19	69.182	<0.001
	4–6g	909	63.26	528	36.74			1,074	74.74	363	25.26		
	7g or above	336	44.56	418	55.44			444	58.89	310	41.11		
Smoking history	No	893	55.02	730	44.98	15.855	<0.001	1,125	69.32	498	30.68	1.051	0.305
	Yes	431	64.04	242	35.96			481	71.47	192	28.53		
Drinking history	No	892	57.25	666	42.75	0.338	0.561	1,106	70.99	452	29.01	2.497	0.114
	Yes	432	58.54	306	41.46			500	67.75	238	32.25		
Exercise weekly	No	882	59.12	610	40.88	4.928	0.085	1,050	70.38	442	29.62	0.816	0.665
	1–3 times	214	57.07	161	42.93			255	68	120	32		
	4–7 times	228	53.15	201	46.85			301	70.16	128	29.84		
rs2472510	TT	623	27.13	421	18.34	4.139	0.126	752	32.75	292	12.72	8.615	0.013
	TG	580	25.26	444	19.34			712	31.01	312	13.59		
	GG	121	5.3	107	4.66			142	6.18	86	3.75		
rs2515614	AA	615	26.79	421	18.34	3.185	0.203	744	32.4	292	12.72	8.084	0.018
	AC	584	25.44	442	19.25			716	31.18	310	13.5		
	CC	125	5.4	109	4.47			146	6.36	88	3.83		
rs2297406	CC	720	31.36	557	24.26	2.621	0.27	884	38.5	393	17.12	1.834	0.4
	CT	522	22.74	366	15.94			624	27.18	264	11.5		
	TT	82	3.58	49	2.13			98	4.26	33	1.44		
rs2472433	CC	415	18.07	293	12.76	1.331	0.514	508	22.13	200	8.71	1.893	0.388
	CT	668	29.09	484	21.08			792	34.49	360	15.68		
	TT	241	10.5	195	8.49			306	13.33	130	5.66		

*SD, standard deviation; IQR, interquartile range; SBP, systolic blood pressure; DBP, diastolic blood pressure; TC, total cholesterol; TG, triglyceride; HDL-C, high-density lipoprotein cholesterol; LDL-C, low-density lipoprotein cholesterol. p < 0.05 is considered statistically significant*.

**Table 3:** Hardy-Weinberg Equilibrium analysis of the ABCA1 genotype.

**SNPs**	**Genotype**	**N**	**MAF**	**χ2**	***P***
rs2472510	TT	1,044	0.32	1.124	0.57
	TG	1,024			
	GG	228			
rs2515614	AA	1,036	0.33	1.169	0.557
	AC	1,026			
	CC	234			
rs2297406	CC	1,277	0.25	2.098	0.35
	CT	888			
	TT	131			
rs2472433	CC	708	0.44	0.736	0.692
	CT	1,152			
	TT	436			

### Association Between ABCA1 Gene Polymorphisms and Hypertension

**Figure 1:**
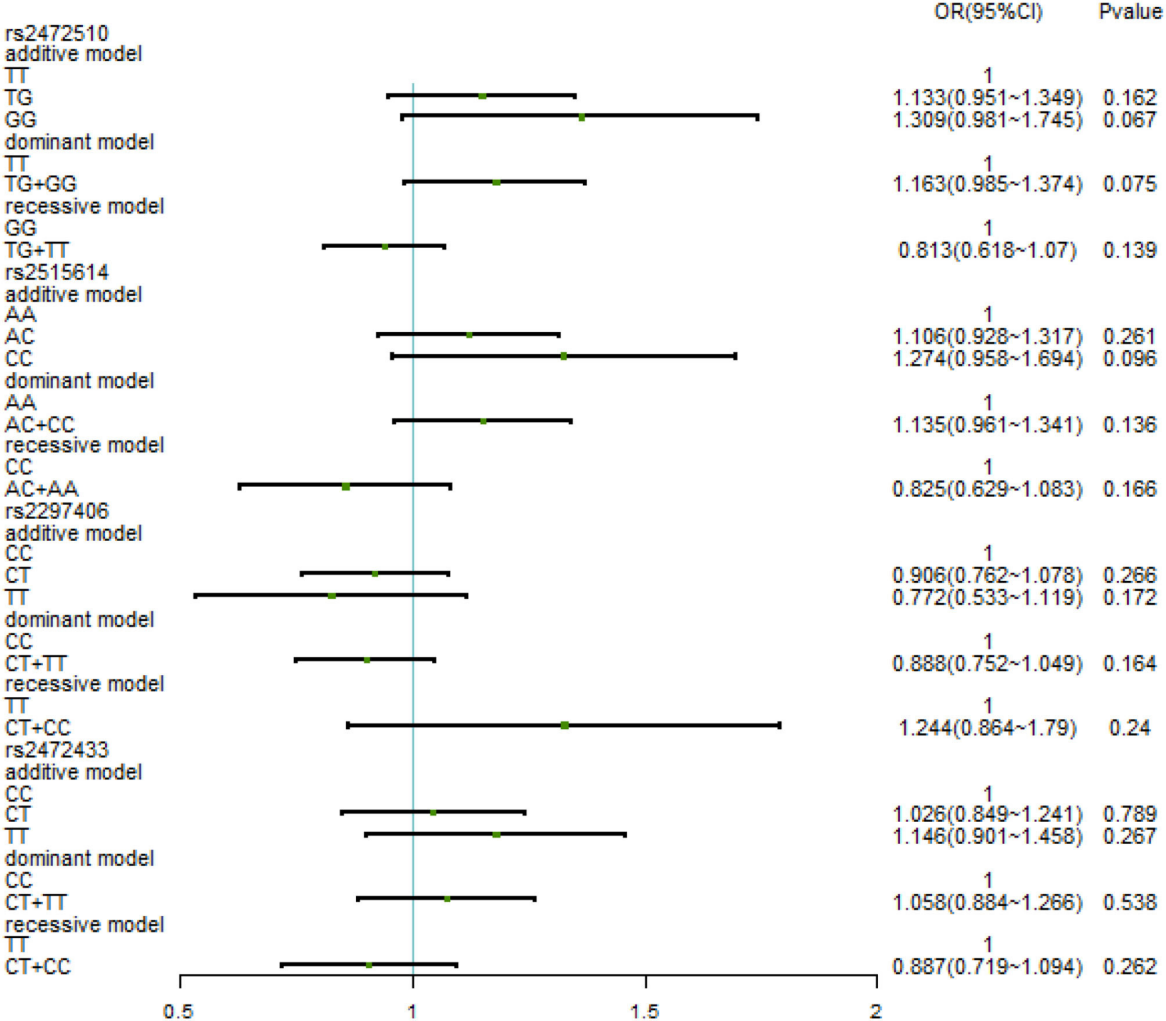
Binary logistic regression analysis for the association between genotypes of rs2472510, rs2515614, rs2297406, rs2472433, and high SBP.

**Figure 2:**
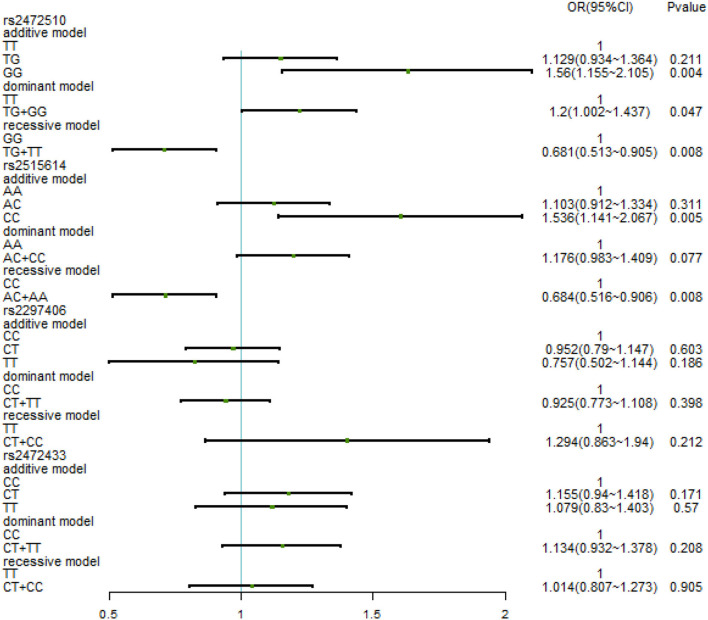
Binary logistic regression analysis for the association between genotypes of rs2472510, rs2515614, rs2297406, rs2472433, and high DBP.

The relationship between different gene models under each ABCA1 locus and hypertension was analyzed by binary logistic regression. From the results shown in Figure 1, there is no relationship between the various genotypes under rs2472510, rs2515614, rs2297406, rs2472433, and high SBP. However, in rs2472510, the GG type (odds ratio [OR] = 1.56, 95% CI = 1.155–2.105, *p* = 0.004) in the additive model and the TG + GG type (OR = 1.2, 95% CI = 1.002–1.437, *p* = 0.047) in the dominant model were more likely to have high DBP as compared to the TT type, and in the recessive model, the TG + TT type (OR = 0.681, 95% CI = 0.513–0.905, *p* = 0.008) had a high risk of DBP, which was 0.681 times than that of the GG type. In rs2515614, the CC type (OR = 1.536, 95% CI = 1.141–2.067, *p* = 0.005) in the additive model was more likely to have high DBP than the AA type, and the AC + AA type (OR = 0.684, 95% CI = 0.516–0.906, *p* = 0.008) in the recessive model had a 0.684 times higher risk of high DBP than the CC type. Neither rs2297406 nor rs2472433 was associated with high DBP (Figure 2).

**Figure 3:**
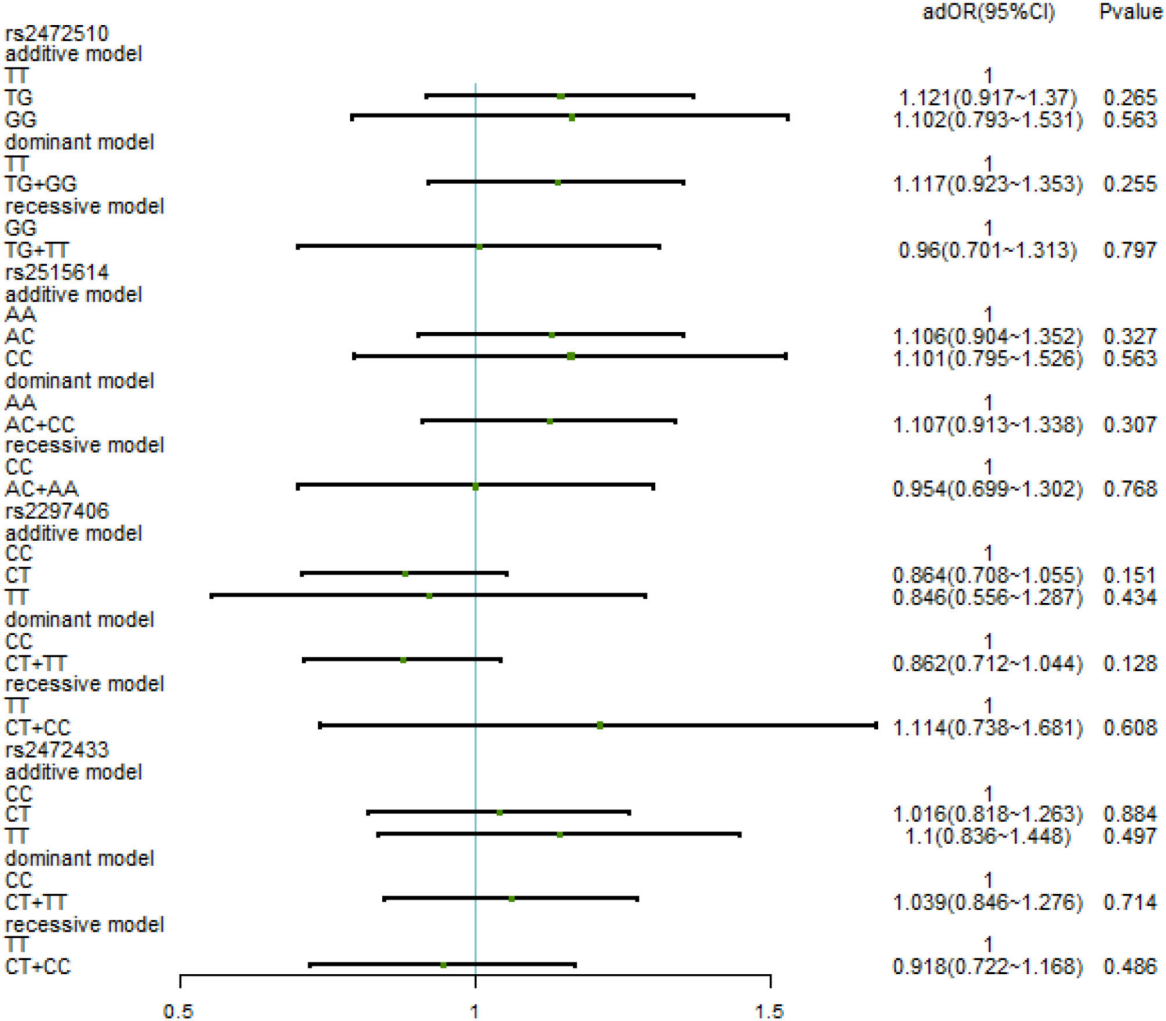
Binary logistic regression analysis for association between genotypes of rs2472510, rs2515614, rs2297406, rs2472433, and high SBP. Adjusted some covariates, such as age, BMI, waist circumference, TC (total cholesterol)/TG (triglyceride)/LDL-C (low-density lipoprotein)/HDL-C (high-density lipoprotein cholesterol), family history, education level, daily iodized salt consumption, and smoking history.

**Figure 4:**
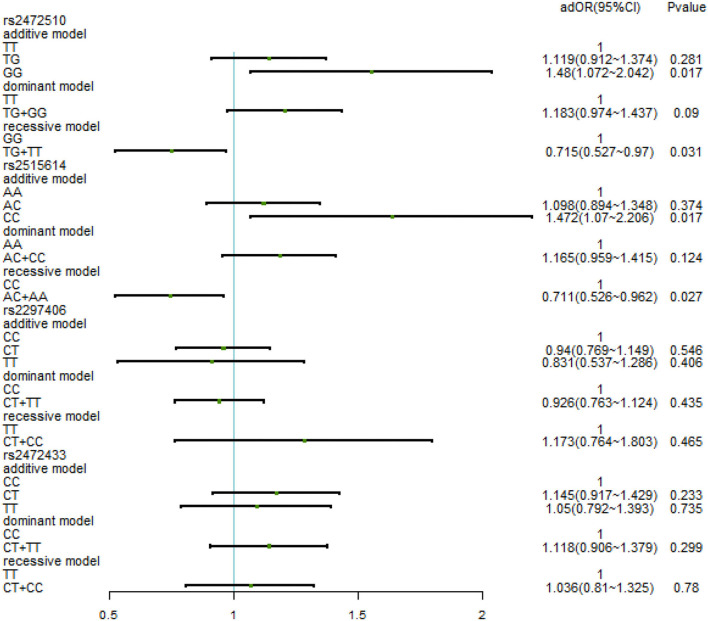
Binary logistic regression analysis for association between genotypes of rs2472510, rs2515614, rs2297406, rs2472433, and high DBP. Adjusted some covariates, gender, age, BMI, waist circumference, TC/TG/LDL-C, family history of the disease, education level, times of fried foods per week, times of desserts per week, and daily iodized salt consumption.

After adjusting for factors associated with high SBP in the previous correlation analysis, such as age, BMI, waist circumference, TC/TG/LDL-C/HDL-C, family history, education level, daily iodized salt consumption, and smoking history, there was still no relationship between the various genotypes under the four SNPs and high SBP (Figure 3). In the high DBP group, adjustments were made for gender, age, BMI, waist circumference, TC/TG/LDL-C, family history of disease, education level, frequency of fried foods per week, times of desserts per week, and daily iodized salt consumption quantity. In rs2472510, the risk of high DBP in the GG type (adOR = 1.48, 95% CI = 1.072–2.042, *p* = 0.017) in the additive model was 1.48 times higher than that of the TT type, and in the recessive model, the risk of high DBP in the TG + TT type (adOR = 0.715, 95% CI = 0.527–0.97, *p* = 0.031) was 0.715 times than that of the GG type, but there was no statistical significance in the dominant model. In rs2615614, the risk of high DBP in the CC type (adOR = 1.472, 95% CI = 1.07–2.206, *p* = 0.017) was 1.472 times higher than that in the AA type in the additive model, while the risk of high DBP in the AC + AA type (adOR = 0.711, 95% CI = 0.526–0.962, *p* = 0.027) was 0.711 times higher than that in the CC type in the recessive model. The dominant genotype was not statistically significant. After adjusting for possible confounders, neither rs2297406 nor rs2472433 remained associated with high DBP (Figure 4).

### Association Between ABCA1 Haplotypes and Hypertension

The frequencies of the four haplotypes were compared between the hypertensive cases and controls, and the allelic arrangement of the haplotypes was rs2297406-rs2472433-rs2472510-rs2515614. Eight common haplotypes (frequency > 1%) that were derived from the four SNPs accounting for 99.6% of the haplotype variation were selected, and the remaining haplotypes were CCGA, TTTC, TCGA, TTGA, TCTC, CCTC, CTTC, and CTGA (the frequencies of the all were less than 1%). Table 4 shows the relationship between haplotypes and high SBP. Taking CTTA as a reference, only the TCTA (OR = 0.791, 95% CI = 0.659–0.951, *p* = 0.006) type had a 0.791 times higher risk of high SBP, and other haplotypes were not associated with high SBP. In the relationship between haplotype and high DBP (Table 5), compared with CTTA, CCTA had a 0.872 times higher risk of DBP, TCTA was 0.801 times, and TTTA was 0.68 times (OR = 0.872, 95% CI = 0.751–1.013, *p* = 0.037; OR = 0.801, 95% CI = 0.656–0.979, *p* = 0.015; OR = 0.68, 95% CI = 0.491–0.943, *p* = 0.011), and other haplotypes had no statistically significant effect on high DBP.

**Table 4:** Frequencies of haplotypes (rs2297406-rs2472433-rs2472510-rs2515614) among cases and controls and association with the risk of SBP.

**Haplotype**	**Frequency**	**Case (*n*%)**	**Control (*n*%)**	**OR (95%CI)**	***P***
CTTA	0.271	535 (27.58)	705 (26.77)	1	
CCTA	0.238	453 (23.35)	637 (24.18)	0.917 (0.799~1.052)	0.109
CCGC	0.129	257 (13.25)	333 (12.64)	1.013 (0.851~1.206)	0.556
TCTA	0.12	210 (10.82)	338 (12.83)	0.791 (0.659~0.951)	0.006
CTGC	0.108	229 (11.80)	266 (10.10)	1.144 (0.948~1.379)	0.92
TCGC	0.07	144 (7.42)	178 (6.76)	1.065 (0.848~1.337)	0.702
TTTA	0.045	82 (4.23)	125 (4.75)	0.85 (0.639~1.131)	0.133
TTGC	0.015	27 (1.39)	40 (1.52)	0.878 (0.537~1.436)	0.301

**Table 5:** Frequencies of haplotypes (rs2297406-rs2472433-rs2472510-rs2515614) among cases and controls and association with the risk of DBP.

**Haplotype**	**Frequency**	**Case (*n*%)**	**Control (*n*%)**	**OR (95%CI)**	***P***
CTTA	0.271	381 (27.69)	858 (26.83)	1	
CCTA	0.238	312 (22.67)	779 (24.36)	0.872 (0.751~1.013)	0.037
CCGC	0.129	187 (13.59)	403 (12.6)	1.044 (0.867~1.126)	0.677
TCTA	0.12	147 (10.68)	400 (12.51)	0.801 (0.656~0.979)	0.015
CTGC	0.108	163 (11.85)	332 (10.38)	1.111 (0.911~1.356)	0.849
TCGC	0.07	108 (7.85)	213 (6.66)	1.143 (0.898~1.454)	0.862
TTTA	0.045	49 (3.56)	158 (4.94)	0.68 (0.491~0.943)	0.011
TTGC	0.015	23 (1.67)	44 (1.38)	1.167 (0.702~1.94)	0.722

## Discussion

This study explored the significant relationship between four SNPs (rs2297406, rs2472433, rs2472510, and rs2515614) in *ABCA1* with hypertension in the southeast Chinese Han population. The evidence suggested that mutations in the *ABCA1* gene were associated with extremely low levels of HDL in humans (20).

High-density lipoproteins (HDL) have a mean size of 8–10 nm and a density of 1.063–1.21 g/ml (21). The biogenesis of HDL and synthesis and secretion of apolipoproteinsA1 (ApoA1) mainly happen in the liver and intestine. ApoA1 interacts with the *ABCA1* expressed by many cell types (including hepatocytes, enterocytes, and macrophages) to exchange lipids producing a nascent HDL particle (22, 23). Therefore*, ABCA1* performs a necessary role in mediating cellular cholesterol, phospholipid efflux, and HDL biosynthesis (24). *ABCA1* was an integral membrane protein, which transported excess cholesterol from peripheral tissues to the liver for excretion, by mediating the first step of reverse cholesterol transport (RCT) (25, 26). Xu et al. verified that cholesterol efflux from monocyte-derived macrophages to autologous serum, HDL was impaired in hypertensive patients, and that *ABCA1* expression was associated with this impairment (17).

Recent studies have reported that *ABCA1* plays a key role in the biogenesis of HDL (17). ABCA1 influenced the secretion of cellular inflammatory cytokine by modulating cholesterol content in the plasma membrane and within intracellular compartments. In humans, the mutations of *ABCA1* can lead to cholesterol deposition in tissue macrophages, resulting in a severe family HDL-deficiency syndrome (20, 27). In addition, *ABCA1* was identified to be the causative gene for Tangier disease (TD), a rare genetic disorder that exhibits severe HDL reduction and a high incidence of premature cardiovascular disease. Therefore, *ABCA1* plays an important function in the development of atherosclerosis and hypertension (28).

It was shown by a mouse model that a specific deletion of *ABCA1* in principal cells would elevate the intracellular cholesterol levels and increase SBP (29). Wang et al. have also demonstrated that inhibitors of *ABCA1* stimulate ENaC in distal renal cells, through a pathway associated with inhibition of the *ABCA1* transporter, increases intracellular cholesterol (30). ENaC plays an important role in regulating sodium reabsorption and controlling BP levels. Therefore, mutations in *ABCA1* lead to intracellular cholesterol accumulation and stimulate ENaC activity, increasing BP levels finally (31).

Furthermore, a study supported that the impairment of ABC transporters might not only potentially influence the cholesterol deposition in monocyte/macrophages to form foam cells but also indicate vascular injury in hypertensive patients (17). Cholesterol accumulation was considered to be associated with the vascular endothelium that actively regulates vascular tone, lipid breakdown, thrombogenesis, inflammation, and vessel growth (32). Hypertension was considered to be related to the inflammatory and immune processes. Therefore, our study suggests that the ABCA1 gene polymorphisms may contribute to the inflammatory responses, vascular injury processes, cholesterol efflux dysfunction, and the activation of the ENaC, ultimately resulting in increased BP levels. These require further research and exploration for us.

The environmental factors of hypertension were also analyzed in this study. We found that age, BMI, waist, dyslipidemia, family history, daily iodized salt consumption, and educational level were associated with hypertension. Aging was a vital contributor to the development of hypertension. It was predicted that the elderly will account for 30% of the whole population of China by 2050. Therefore, regularly monitoring BP levels in middle-aged and elderly people was very significant (33). BMI and waist-to-hip ratio had been a comprehensive indicator of the outcome of acquired lifestyle and are closely associated with the occurrence of hypertension (34, 35). Family history of hypertension was a crucial marker of genetic factors, it was used as an alternative indicator to study the relationship between genetic factors and diseases frequently (36). The speedy economic growth, especially with a higher dietary salt intake, was another new and crucial factor related to the increased prevalence of hypertension in China, and quite a few lines of proof that include epidemiological observations, animal studies, and clinical trials have constantly proven a causal relationship between dietary salt consumption and hypertension (37, 38). Excess dietary salt consumption was also an essential risk factor for the development and progression of cardiovascular disease. Educational degree is an important socioeconomic factor, there was developing proof that in addition to these widespread risk factors, income level and social status were factors that may associated be with BP control (39). As a result, stopping unhealthy lifestyles may additionally make a contribution to patients' health. Interactions between environmental and epigenetic elements need to be further investigated. Thus, healthcare providers were cautioned to provide furnish extra support and health education for those patients to achieve proposed BP goals and thereby decrease future cardiovascular risk.

There were some limitations to this study. First of all, this study only mentioned the association of ABCA1 gene polymorphisms with hypertension. There were still many unmeasured genetic factors and environmental factors that were not taken into consideration. We could not estimate all the potential risk factors for hypertension completely. Secondly, this study was performed in eastern China, and a massive number of samples were needed to investigate the relationship and confirm our findings. Finally, this study only included Han Chinese people, future research should also encompass different nations or races due to genetic heterogeneity among different human species, this could enable more accurate results.

Despite these limitations, these results still have practical implications for the prevention and treatment of hypertension. Hypertension is one of the most prevalent diseases and public health problems that threaten human health, genetic and genomic research studies have provided important viewpoints into the etiology and pathogenesis of hypertension. Since 2007, the sample size of a series of sequential genome-wide association studies (GWAS) for BP and hypertension has grown exponentially, with the latest recent study involving 1 million subjects and having identified over 1,477 SNPs associated with BP traits, explaining approximately 27% of the 30–50% estimates heritability of BP (40, 41). Because of the complexity of BP regulation mechanisms, further genomic studies may be able to provide genetic tools in the etiology of hypertension and provide a new treatment option for hypertension patients. In addition, genomics can provide a deeper understanding of molecular pathways that regulate BP, which can inform precision medicine and provide the basis for new drug development and personalized treatment.

## Conclusion

In conclusion, the present study has identified that hypertension probability was associated with rs2472510 and rs2515614. Considering these relevant factors can also show informative for predicting hypertension.

## Data Availability Statement

The datasets presented in this study can be found in online repositories. The names of the repository/repositories and accession number(s) can be found in the article/Supplementary Material.

## Ethics Statement

The studies involving human participants were reviewed and approved by the Medical Ethics Committee of Hangzhou Normal University (No. 2013020). The patients/participants provided their written informed consent to participate in this study.

## Author Contributions

YR, ET, XT, and LY designed the study and with all co-authors carrying out the study design. CD, YZ, and LX recruited and followed up the study participants. YR and ET drafted the manuscript and which was revised by all authors. All authors read and approved the final manuscript.

## Funding

This study was supported by grants from the Natural Science Foundation of Zhejiang Province (Grant Nos. LQ18H190003 and LY12H16028) and the National Natural Science Foundation of China (Grant No. 81772168).

## Conflict of Interest

The authors declare that the research was conducted in the absence of any commercial or financial relationships that could be construed as a potential conflict of interest.

## Publisher's Note

All claims expressed in this article are solely those of the authors and do not necessarily represent those of their affiliated organizations, or those of the publisher, the editors and the reviewers. Any product that may be evaluated in this article, or claim that may be made by its manufacturer, is not guaranteed or endorsed by the publisher.
