# Supplementary material for: Association Between ABCA1 Gene Polymorphisms and the Risk of Hypertension in the Chinese Han Population
Source: Front Public Health. 2022 May 20;10:878610. doi: 10.3389/fpubh.2022.878610 (PMC9163321; doi:10.3389/fpubh.2022.878610)
Supplement: Supplementary file 1 [file Table_1.docx]

| Appendix Table1. Sequences of primers and probes used for genotyping | | |
| --- | --- | --- |
| SNPs | Primers for PCR | Probes for detecting variation |
| rs2472510 | F:CTGACATTAGGCAAGTTGCTG | TA:TTTTTTTTTTTTTTTTCCTATAGTGAAATTTATTCATACGA |
|  | R:ATTATAGGCAGAAGCCATCGC | TC:TTTTTTTTTTTTTTTTTTTCCTATAGTGAAATTTATTCATACGC |
|  |  | TR:-P-GTGTTTTCAGTGATTTTAAAAGCTTGCTTTTTTTTTTT-HEX- |
| rs2515614 | F:ATTCCAACAATGGAGGAGCAC | TG:TTTTGTTGGTACAAAACCCTTTAGCTTTG |
|  | R:CAACTTAGTGATGGGAAGGAG | TT:TTTTTTTGTTGGTACAAAACCCTTTAGCTTTT |
|  |  | TR:-P-GCAAACCTCCTTTAAGACCCGATTTTTT-HEX- |
| rs2297406 | F:ATTGCCATGAACTTGGCCTTC | TC:TTTTTTTTGCCAGGCCCTCACAGCTATACCAAC |
|  | R:ACCAGCCCATAGTAAGGTTTG | TT:TTTTTTTTTTTGCCAGGCCCTCACAGCTATACCAAT |
|  |  | TR:-P-CAAATCAGTTCTTACAACCAGTCCCTTTTTT-FAM- |
| rs2472433 | F:GACTAGTGTCACAAAGCTCAG | TA:TGGACGAGATCACTTCAAAGGTCTA |
|  | R:CACCACGCTGAGCTAATTTTC | TG:TTTTGGACGAGATCACTTCAAAGGTCTG |
|  |  | TR:-P-TAATCCCACGTCTATAATCCCAGCA-FAM- |
|  |  |  |
